# Respiratory pathogens detected in specimens collected for COVID-19 surveillance in Zambia

**DOI:** 10.4102/jphia.v16i1.684

**Published:** 2025-02-16

**Authors:** Martin Nyahoda, Ngonda Saasa, Katendi Changula, Walter Muleya, Zachariah Mupila, Chilufya Chikoti, Gift Moonga, Catherine Sutcliffe, Geoffrey Kwenda, Edgar Simulundu

**Affiliations:** 1 Department of Disease Control, School of Veterinary Medicine, University of Zambia, Lusaka, Zambia; 2 Department of Paraclinical Studies, School of Veterinary Medicine, University of Zambia, Lusaka, Zambia; 3 Microbiology Laboratory, University Teaching Hospital, Lusaka, Zambia; 4 Department of Epidemiology, Bloomberg School of Public Health, John Hopkins University, Baltimore, United States of America; 5 Department of Biomedical Sciences, School of Health Sciences, University of Zambia, Lusaka, Zambia

**Keywords:** respiratory pathogens, COVID-19, co-infections, surveillance, Zambia

## Abstract

**Background:**

In Zambia, knowledge on the landscape of respiratory pathogens that circulated during the coronavirus disease 2019 (COVID-19) pandemic is limited.

**Aim:**

This study investigated respiratory pathogens that circulated in Zambia during the COVID-19 pandemic.

**Setting:**

Nasopharyngeal specimens collected between July 2020 and July 2021 for COVID-19 testing in hospitals, ports of entry, persons seeking certificates for international travel and in communities were used.

**Methods:**

Proportional age-stratified sampling was used to select 128 specimens. The samples were screened for 33 other respiratory pathogens using the Fast Track Diagnostics multiplex molecular assay.

**Results:**

Overall, 71.1% (*n* = 91/128) tested positive for at least one respiratory pathogen. Bacterial respiratory pathogens were more predominant (70.3%, *n* = 90/128) than viral (51.6%, *n* = 66/128). *Staphylococcus aureus* was the most prevalent, detected in 22.7% (*n* = 29/128). The prevalence of influenza was 13.3% (*n* = 17/128). Rhinovirus had a prevalence of 3.1% (*n* = 4/128), while it was 10.1% (*n* = 13/128) for adenovirus. Children, adolescents and the elderly accounted for most influenza-positive specimens, 76.5% (*n* = 13/17), while 100% (*n* = 3/3) of specimens positive for *Moraxella catarrhalis* were from children. All specimens testing positive for *Haemophilus influenzae*, 100% (*n* = 5/5) were from children and adolescents. Co-infections were detected in 57.1% (*n* = 52/91) of specimens testing positive for at least one pathogen.

**Conclusion:**

Bacterial respiratory pathogens appeared to predominate circulation during the COVID-19 pandemic period.

**Contribution:**

Bacterial respiratory pathogens should not be neglected when implementing public health mitigation measures.

## Introduction

Zambia reported the first polymerase chain reaction (PCR)-confirmed coronavirus disease 2019 (COVID-19) case on 16 March 2020.^1^ By 18 April 2020, at least 100 COVID-19 cases were reported in the country.^2^

With the emergence of COVID-19, the Zambia National Public Health Institute (ZNPHI) led the country’s COVID-19 pandemic response with surveillance being a core activity. This included collecting nasopharyngeal (NP) specimens at ports of entry, contact tracing, health worker testing and health facility inpatient screening from both symptomatic and asymptomatic persons. Additionally, like in many other countries, a wide range of non-pharmaceutical interventions (NPIs) were implemented to limit the spread of the virus. These measures included physical distancing, handwashing, face masking, respiratory etiquette, travel restrictions, remote working and school closures.^3,4,5^

Studies have shown that NPIs implemented against COVID-19 have had an impact on the transmission dynamics of respiratory pathogens.^6^ Epidemiological studies have shown a marked decrease in influenza and other respiratory infections during the COVID-19 pandemic. A study conducted in Japan using data from 2014 to 2020 revealed that influenza PCR-confirmed cases dropped in the 2019–2020 season by about 50% in persons less than 15 years of age with a similar trend being observed in other age groups.^4^ In China, it was observed that the influenza season abruptly ended resulting in the season being shorter by 9 weeks.^7^ The decrease in influenza activity was observed despite an increase in specimens tested from an average of 4104 to 5711 per week.^7^ In Hong Kong, influenza community transmissions decreased by 44%, while paediatric hospitalisation decreased by 33% with the effective reproductive number dropping from 1.28 before the COVID-19 pandemic to 0.72 after emergence of the pandemic.^6^ In the United States (US), samples testing positive for influenza decreased from a median of 19.24% to 0.33% with a similar trend being observed in Australia, Chile and South Africa.^8^

Similarly, a significant decrease in infections for other respiratory pathogens of concern such as respiratory syncytial virus (RSV), parainfluenza viruses, human rhinovirus (HRV) and enterovirus was observed during the pandemic period.^9^ In England, confirmed cases of RSV hospital admissions and emergency attendances were substantially reduced by over 80% in children aged 5 years and below.^10^ In Japan, no RSV outbreak was reported in 2020 at the peak of the pandemic, while there was a 93.4% reduction of RSV infections in Australia.^11^ This trend was similar with other non-enveloped respiratory viruses such as enteroviruses, adenoviruses and human bocavirus. Detection of these non-enveloped viruses declined as with enveloped respiratory viruses globally.^12^ The modifications in the circulation of bacterial respiratory pathogens were also observed during the pandemic as with viral respiratory pathogens.^12^

Information on changes in the epidemiology of respiratory pathogens in Zambia during the COVID-19 pandemic is limited, with only a few reports from rural Zambia.^13,14^ In this study, we investigated the prevalence of a broad range of respiratory pathogens circulating in Zambia during the COVID-19 pandemic using specimens that were collected from both rural and urban areas for severe acute respiratory syndrome coronavirus 2 (SARS-CoV-2) testing.

## Research methods and design

### Study design

This was a retrospective study using NP specimens collected for COVID-19 testing across various provinces of Zambia from both rural and urban areas. For the purpose of this study, proportional age-stratified sampling was used to select specimens to build a convenient sample (*n* = 128), consisting of 88 from urban and 40 from rural areas (Table 1). The samples were drawn from stored residual specimens collected between July 2020 and July 2021.

**TABLE 1:** Demographic and geographic characteristics of selected specimens.

S/N	Age group (years)	Place of collection (town, province)	Geographical classification	Specimens selected	Age group (Total)
1	0–9	Lusaka, Lusaka	Urban	23	26
		Isoka, Muchinga	Rural	1	
		Mansa, Luapula	Rural	2	
2	10–19	Lusaka, Lusaka	Urban	19	34
		Isoka, Muchinga	Rural	2	
		Mansa, Luapula	Rural	13	
3	20–64	Mansa, Luapula	Rural	10	44
		Isoka, Muchinga	Rural	4	
		Chinsali, Muchinga	Rural	3	
		Lusaka, Lusaka	Urban	27	
4	≥ 65	Mungwi, Northern	Rural	1	24
		Chinsali Muchinga	Rural	1	
		Mansa, Luapula	Rural	1	
		Lusaka, Lusaka	Urban	21	

**Totals**	**-**	**-**	**-**	**128**	**128**

S/N, serial number.

### Study setting and data collection

The University of Zambia, School of Veterinary Medicine, Department of Disease Control served as a key testing centre for COVID-19 throughout the pandemic and provided PCR-based confirmation for the first case on 16 March 2020. Specimens collected by ZNPHI from all provinces of the country between 2020 and 2021 were submitted to the university for testing. These specimens were collected as part of surveillance at various health facilities, ports of entry, during contact tracing efforts and mass community screening as well as from persons seeking medical certificates for international travel. After testing for SARS-CoV-2, residual specimens were anonymised and stored at –80 °C.

### Sampling strategy

The specimens were disaggregated by age, sex, year of collection, geographical location (rural or urban) and then stratified into age groups comprising of children 0–9 years, adolescents 10–19 years, adults 20–64 years and the elderly aged ≥ 65 years (Table 1). Specimens were then randomly selected from these age groups to make a total sample size of 128. The sample size was informed by the quantity of available testing reagents. Only specimens that were clearly identified with required demographic information such as age, date and place of collection were included.

### Nucleic acid extraction

Nucleic acid extraction was performed using NucliSENS^®^ easyMAG^®^ (bioMerieux, Durham, US) according to the manufacturer’s instructions. On completion of the automated extraction process, 110 µL of nucleic acid was eluted and stored at –80 °C until required for further analysis.

### Detection of respiratory pathogens

For COVID-19 testing, ribonucleic acid (RNA) extraction and molecular detection were performed as previously described.^1^ To detect influenza and other respiratory pathogens, the fast track diagnostics (FTD) respiratory pathogens 33 (FastTrack Diagnostics, Luxembourg) multiplex rRT-PCR test kit was used on a QuantStudio^®^ 5 (Life Technologies, Singapore) thermocycler, following the manufacturer’s instructions. The assay detects 33 respiratory pathogens as follows: influenza A virus (IAV), influenza A(H1N1) virus (swine lineage) (IAV[H1N1] swl), influenza B virus (IBV), influenza C virus (IVC), human coronaviruses (HCoV) NL63, 229E, OC43 and HKU1, human parainfluenza viruses (HPIV) 1, 2, 3 and 4, human metapneumoviruses (HMPV) A and B, HRV, human respiratory syncytial viruses (HRSV) A and B, human adenovirus (HAdV), enterovirus (EV), human parechovirus (HPeV), human bocavirus (HBoV), *Pneumocystis jirovecii, Mycoplasma pneumoniae, Chlamydophila pneumoniae, Streptococcus pneumoniae, Haemophilus influenzae* B, *Staphylococcus aureus, Moraxella catarrhalis, Bordetella* spp. (except *Bordetella parapertussis*), *Klebsiella pneumoniae, Legionella pneumophila, Legionella longbeachae, Salmonella* spp., *Haemophilus influenzae* and equine arteritis virus (EAV), which serves as an internal control (IC). The thermocycler was programmed and optimised for fast-track master mix PCR based on the manufacturer’s recommendations as follows: 50 °C for 15 min, 94 °C for 1 min, 45 cycles of 94 °C for 8 s and 45 cycles of 60 °C for 1 min.

### Data analysis

QuantStudio^®^ Design and Analysis software version 2.6.0 (Thermo Fisher Scientific, Waltham, US) was used for analysis of the presence and absence of target pathogens. Presence or absence of target pathogens was determined using fluorescence signals for reporter dyes. Amplification plots were considered positive for the target pathogen when the cycle threshold (Ct) values did not exceed 35. Logistic regression in Statistical Package for Social Sciences (SPSS) (version 20, IBM Corp, 2011) was performed adjusting for age and location (rural or urban). Data management for profiling of co-infections was performed in Microsoft Excel.

### Ethical considerations

Ethical clearance and waiver of consent was obtained from Macha Research Trust Institutional Review Board, approval number (E2021.05). Approval to conduct the study was granted by Zambia National Health Research Authority, approval number (NHRA000042/30/03/2022). To protect participant information, all specimens were de-identified by removing mobile telephone numbers, residential addresses and names.

## Results

### Prevalence of respiratory pathogens

**FIGURE 1:**
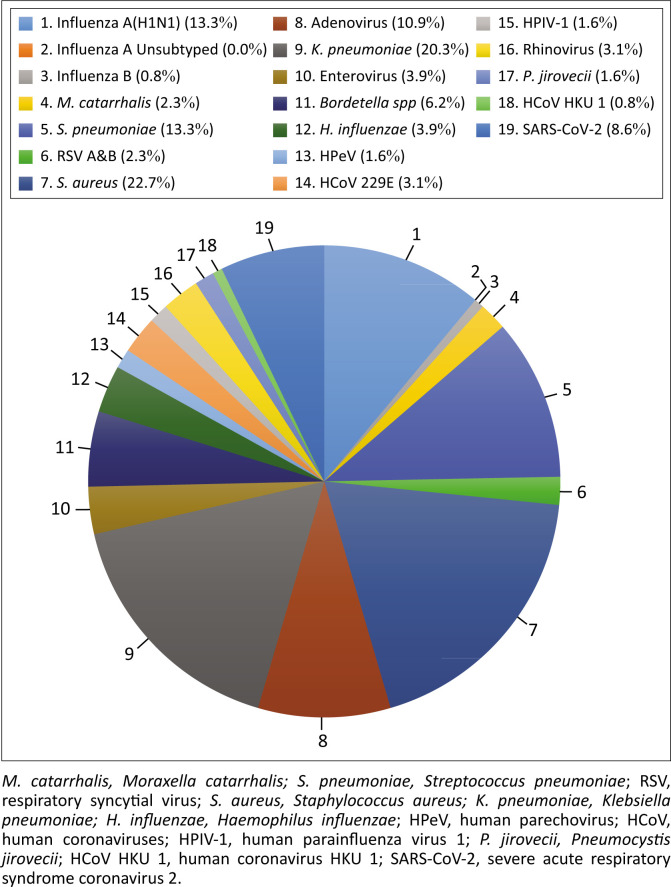
Percentage distribution of detected respiratory pathogens.

From all 128 specimens tested, 71.1% (*n* = 91/128) were positive for at least one respiratory pathogen. Overall, bacterial respiratory pathogens were more prevalent in 70.3% (*n* = 90/128) than viral respiratory pathogens in 51.6% (*n* = 66/128). *Staphylococcus aureus* was the most detected respiratory pathogen with a prevalence of 22.7% (n = 29/128), followed by *Klebsiella pneumoniae* that accounted for 20.3% (*n* = 26/128) (Figure 1). *Streptococcus pneumoniae* and *Bordetella pertussis* had a prevalence of 13.3% and 6.2%, respectively. Influenza A(H1N1) accounted for 13.3% (*n* = 17/128), while influenza B accounted for only 0.8% (*n* = 1/128). Coronaviruses excluding SARS-CoV-2 accounted for 3.9% (*n* = 5/128). Human adenovirus accounted for 10.9% (*n* = 14/128), while enterovirus had a prevalence of 3.9% (*n* = 5/128).

### Rural-urban geographical distribution of pathogens

Except for *S. pneumoniae,* the prevalence of bacterial pathogens was higher in rural areas than in urban areas (Table 2). *Staphylococcus aureus* had a rural prevalence of 27.5% compared to 20.5% in urban areas. *Bordetella pertussis* had a rural prevalence of 10% compared to 4.5% for urban.

**TABLE 2:** Distribution of pathogens by rural-urban geographic classifications.

Pathogen	Urban specimens positive	Rural specimens positive	% Prevalence urban (*n* = 88)	% Prevalence rural (*n* = 40)
Influenza A(H1N1)	12	5	13.6	12.5
Influenza B	1	0	1.1	0
*M. catarrhalis*	2	1	2.3	2.5
*S. pneumoniae*	10	3	11.4	7.5
RSV A&B	3	0	3.4	0
*S. aureus*	18	11	20.5	27.5
Adenovirus	11	3	12.5	7.5
*K. pneumoniae*	17	9	19.3	22.5
Enterovirus	5	0	5.7	0
*Bordetella spp*	4	4	4.5	10
*H. influenzae*	5	0	5.7	0
HPeV	1	1	1.1	2.5
HCoV 229E	5	0	5.7	0
HPIV-1	2	0	2.3	0
Rhinovirus	4	0	4.5	0
*P. jirovecii*	1	1	1.1	2.5
HCoV HKU 1	0	1	0.0	2.5
SARS-CoV-2	10	1	11.4	2.5

*M. catarrhalis, Moraxella catarrhalis; S. pneumoniae, Streptococcus pneumoniae*; RSV, respiratory syncytial virus; *S. aureus, Staphylococcus aureus; K. pneumoniae, Klebsiella pneumoniae*; HPeV, human parechovirus; HPIV-1, human parainfluenza virus 1; *P. jirovecii, Pneumocystis jirovecii*; SARS-CoV-2, severe acute respiratory syndrome coronavirus 2; HCoV, human coronaviruses.

Viral respiratory pathogens were more prevalent in urban areas compared to rural areas. All the specimens that tested positive for rhinovirus 100% (*n* = 4/4) and RSV 100% (*n* = 3/3) were from urban areas. Of the total 11 specimens testing positive for SARS-CoV-2, only 9.1% (*n* = 1/11) was from a rural area, while the rest 90.9% (*n* = 10/11) were from urban areas. Results of logistic regression showed that adjusted odds ratios associated with location, rural or urban were 3.10 for *Bordetella pertussis* confidence interval (CI) (0.667–14.208), 1.362 for *S. aureus* CI (0.561–3.303) and 1.588 for *K. pneumoniae* CI (0.615–4.097). The majority of other pathogens had odds ratios < 1.

### Distribution of respiratory pathogens by age group

**FIGURE 2:**
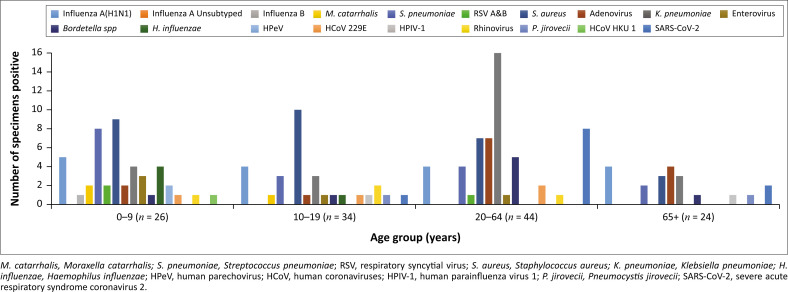
Distribution of respiratory pathogens by age group.

Bacterial respiratory pathogens were mostly detected in children and the elderly (Figure 2). *Moraxella catarrhalis* was only detected in children and adolescents (*n* = 3/3) with no specimens from adults and the elderly testing positive for the bacteria. *Staphylococcus aureus* was detected in specimens from all age groups with children and adolescents accounting for 65.5% (*n* = 19/29), while adults and the elderly accounted for 24.1% and 10.3%, respectively. Of the total eight specimens testing positive for *Bordetella pertussis*, 25% (*n* = 2/8) were from children and adolescents, while 75% (*n* = 6/8) were from adults and the elderly. *Klebsiella pneumoniae* was mostly detected in adults and the elderly accounting for 73.1% (*n* = 19/26), while children and adolescents accounted for 26.9%. All specimens testing positive for *Haemophilus influenzae* (100%, *n* = 5/5) were from children and adolescents. Similarly, except for SARS-CoV-2, viral respiratory pathogens were mostly detected in specimens from children and adolescents compared to other age groups. Influenza A(H1N1) was mostly detected in children, adolescents and the elderly accounting for 76.5% (*n* = 13/17) of the total 17 specimens testing positive for influenza, while adults accounted for 23.5% (*n* = 4/17) of positive specimens. Only one specimen was positive for SARS-CoV-2 in children and adolescents representing 9.1%, while the majority, that is, 90.9% (*n* = 10/11) were all from adults and the elderly.

### Co-infections of respiratory pathogens

Overall, co-infections were observed in 57.1% (*n* = 52/91) of specimens testing positive for at least one respiratory pathogen. The majority of co-infections were observed in children and adolescents (44.2%; *n* = 23/52). Of the co-infections observed, 11.5% (*n* = 6/52) specimens had virus-virus co-infections, with the most co-infecting virus being human adenovirus. On the other hand, bacteria-bacteria co-infections were 26.9% (*n* = 14/52) with the most co-infecting bacteria being *S. aureus* (Table 3). Virus-bacteria co-infections accounted for the remaining 48.1% (*n* = 25/52) of co-infections. Severe acute respiratory syndrome coronavirus 2 co-infections were mainly with *S. aureus* 27.3% (*n* = 3/11), *Klebsiella pneumoniae* 27.3% (*n* = 3/11) and *Bordetella pertussis* 18.2% (*n* = 2/11). Only one specimen positive for SARS-CoV-2 was co-infected with RSV, while 18.2% (*n* = 2/11) were co-infected with influenza A(H1N1). Five out of 17 specimens testing positive for influenza A(H1N1) representing 29.4% were co-infected with *S. aureus*, while co-infection with *Streptococcus pneumoniae* was 23.5% (*n* = 4/17). Co-infection of influenza A(H1N1) with *Klebsiella pneumoniae, B. pertussis* and adenovirus was at 11.8% (2/17) for each of these bacteria.

**TABLE 3:** Co-infection profile for respiratory pathogens during the COVID-19 pandemic.

Pathogen	IAV	IBV	*M. Cat*	*S. Pne*	RSV	*S. Aur*	Aden	Enter	*Bord*	*H. Inf*	HPeV	CoV229	HPIV-1	Rhinov	*P. Jir*	HKU 1	S-CoV-2
IAV	X	1	0	1	0	4	5	2	1	1	0	1	0	0	2	0	2
IBV	-	X	0	0	0	0	0	0	0	0	0	0	0	0	0	0	0
*M. Cat*	-	-	X	1	0	0	0	0	0	0	0	0	0	0	0	0	0
*S. Pne*	-	-	-	X	1	5	1	0	0	2	1	2	0	2	0	0	1
RSV	-	-	-	-	X	1	0	0	0	0	1	0	0	0	0	0	1
*S. Aur*	-	-	-	-	-	X	2	2	3	3	0	0	1	1	1	0	3
Aden	-	-	-	-	-	-	X	1	2	1	0	0	0	0	0	0	1
Enter	-	-	-	-	-	-	-	X	0	0	0	0	0	0	0	0	0
*Bord*	-	-	-	-	-	-	-	-	X	1	0	0	0	1	0	0	2
*H. Inf*	-	-	-	-	-	-	-	-	-	X	0	0	0	1	0	0	0
HPeV	-	-	-	-	-	-	-	-	-	-	X	1	0	0	0	0	0
CoV229	-	-	-	-	-	-	-	-	-	-	-	X	0	1	0	0	0
HPIV-1	-	-	-	-	-	-	-	-	-	-	-	-	X	0	0	0	0
Rhinov	-	-	-	-	-	-	-	-	-	-	-	-	-	X	0	0	0
*P. Jir*	-	-	-	-	-	-	-	-	-	-	-	-	-	-	X	0	0
HKU 1	-	-	-	-	-	-	-	-	-	-	-	-	-	-	-	X	0
S-CoV-2	-	-	-	-	-	-	-	-	-	-	-	-	-	-	-	-	X

Note: X = co-infection already ascertained.

IAV, influenza A(H1N1) virus; IBV, influenza B virus; *H. inf, Haemophilus influenzae; M. cat, Moraxella catarrhalis;* CoV229, Human Coronavirus 229E; *Bord, Bordetella pertussis; S. Pne, Streptococcus pneumoniae;* HPIV 1, human parainfluenza virus 1; RSV, respiratory syncytial virus; Rhinov, rhinovirus; *S. aur, Staphylococcus aureus; P. Jir, P. jirovecii;* Aden, human adenovirus; HKU 1, human coronavirus HKU 1; Enter, enterovirus; S-CoV-2, severe acute respiratory syndrome coronavirus 2 (SARS-CoV-2).

## Discussion

The heightened country-wide COVID-19 surveillance efforts following the detection of the first SARS-CoV-2 case in Zambia provided a great opportunity to understand the broad range and epidemiology of respiratory pathogens circulating in Zambia during the pandemic period. Previous studies on respiratory infections were limited in geographic coverage, thus providing limited information on the distribution and burden of respiratory infections in the country.^15,16^ Although most respiratory pathogens of public health concern continued to circulate, we found that bacterial respiratory pathogens were more predominant than viral pathogens during the pandemic period. *Staphylococcus aureus* was the highest detected bacteria pathogen followed by *Klebsiella pneumoniae*. Influenza A(H1N1) was the most prevalent viral respiratory pathogen detected followed by human adenovirus. Bacterial respiratory pathogens seem to have persisted more than viral counterparts during the COVID-19 pandemic despite the implementation of public health and social measures.

Although on observation, it seems that rural geographical areas increased the risk of infection for *Bordetella pertussis* with adjusted odds ratio of 3.10, *S. aureus* 1.362 and 1.588 for *K. pneumoniae*, the confidence intervals > 1 could not statistically support the significance of the observed differences between urban and rural areas.

The results of this study are comparable to other studies^17,18,19^ that have shown a high prevalence of bacterial respiratory infections among hospitalised children, adults and the elderly because children may be immunologically naïve while the elderly may be progressing towards immunosenescence. Existing scientific literature shows that more emphasis has been placed on the role of viral pathogens as aetiological agents of acute lower respiratory tract infections in developing countries neglecting the role of bacterial pathogens.^20,21,22^ While our study could not ascertain whether bacterial pathogens detected caused infections in the individuals tested, at the very least, it does show the presence or colonisation of NP areas with bacteria that could cause respiratory infections and progress to severe disease.

Although we have provided estimates of prevalence for a broad range of respiratory pathogens during the COVID-19 pandemic, it was difficult to determine the extent to which NPIs against COVID-19 had altered the epidemiology of these respiratory pathogens because studies before the pandemic^15,16^ were based on specimens collected entirely from symptomatic individuals in health facilities, thus posing challenges in making comparisons. However, the percentage of specimens testing positive for bacterial and viral pathogens from this study was generally lower than those reported in pre-pandemic studies.^23^ For example, we detected influenza virus, rhinovirus and RSV in 13.3%, 3.1% and 2.3% of the specimens, respectively, while influenza, rhinovirus and RSV among symptomatic outpatients in rural Zambia in 2019 were estimated at 21.5%, 24.0% and 16.5%, respectively.^12^ Similarly, another study conducted to identify viral and bacterial pathogens from hospitalised children with acute respiratory illness in Lusaka between 2011 and 2012 reported an estimated RSV prevalence of 33.7% and rhinovirus at 11.4%.^13^ With regard to bacterial pathogens, this study reported a prevalence of *Streptococcus pneumoniae* at 13.3%, *Moraxella catarrhalis* at 2.3% and *Bordetella pertussis* at 6.2%. On the other hand, a study conducted before the pandemic reported a higher prevalence of *Streptococcus pneumoniae* at 54.8% and *Moraxella catarrhalis* at 46.2% showing a considerable reduction in prevalence.^16^ It is possible that public health and social measures implemented against COVID-19 may have altered the epidemiology of other respiratory pathogens besides helping to slow the transmission of SARS-CoV-2.^29,30,31,32^

Co-infections detected in this study were mostly virus-bacteria and bacteria-bacteria. In a study of virus-virus interactions for respiratory pathogens, it was found that virus-virus interactions may result in interaction-induced effects such as cross-immunity and resource competition leading to attenuation.^20,24^ Additionally, infection with one respiratory pathogen may elicit immune response against the other. This may help to explain the small number of virus-virus co-infections and a higher bacterial co-infections observed in this study. Further, possible bacterial colonisation of the respiratory system may also have contributed to a higher bacteria detection. Largely, co-infections were detected in children and adolescents who accounted for 44.2% (*n* = 23/52) of the total co-infections detected. The results agree with findings of other epidemiological studies in Zambia and globally that have demonstrated that co-infections for respiratory viruses largely occur in children.^16,25,26,27,28^ The most co-infecting respiratory pathogens across all age groups were *Staphylococcus aureus, Streptococcus pneumoniae* and *Klebsiella pneumoniae*. This is the first study in Zambia to show *Staphylococcus aureus* among the most co-infecting respiratory pathogens in Zambia.

It is important to note that recent studies have shown that co-infection with *Staphylococcus aureus* is associated with the highest intensive care admission and death rates.^17,18^ It has further been found that interactions between viruses and bacteria could generally lead to enhanced bacteria binding, immune response dysregulation and slowed bacterial clearance.^17^ Therefore, there is need for epidemiological studies to fully ascertain the association between virus-bacteria co-infection and severe respiratory illnesses leading to hospitalisation and death in Zambia given that previous studies have been more focused on viral pathogens.

This study had limitations including being retrospective study in nature and having a limited sample size. A lack of clinical data on participants contributed to the inability of the study to elucidate associations between co-infections and disease outcomes. There were more specimens collected from urban than rural areas that made the sample size to have a bias towards urban areas.

## Conclusion

This is the first study combining both urban and rural diagnostic specimens to detect respiratory pathogens during the COVID-19 pandemic in Zambia. While both viral and bacterial respiratory pathogens of public health concern continued to circulate during the period, bacterial pathogens were more predominant. Overall, there was an observed decline in the prevalence of respiratory pathogens suggesting that public health interventions against COVID-19 may have altered the epidemiology of respiratory pathogens. Co-infections detected were mostly virus-bacteria with *S. aureus* being the most co-infecting pathogen. Considering the findings from this and other studies, sustaining public health and social measures instituted against COVID-19 may possibly help to limit the spread of other respiratory pathogens of public health concern.
